# Removal of Arterial Vessel Contributions in Susceptibility-Weighted Images for Quantification of Normalized Visible Venous Volume in Children with Sickle Cell Disease

**DOI:** 10.1155/2017/5369385

**Published:** 2017-08-28

**Authors:** Adam M. Winchell, Ruitian Song, Ralf B. Loeffler, Winfred C. Wang, Jane S. Hankins, Kathleen J. Helton, Claudia M. Hillenbrand

**Affiliations:** ^1^Department of Diagnostic Imaging, St. Jude Children's Research Hospital, Memphis, TN, USA; ^2^Department of Hematology, St. Jude Children's Research Hospital, Memphis, TN, USA

## Abstract

**Purpose:**

To evaluate a new postprocessing framework that eliminates arterial vessel signal contributions in the quantification of normalized visible venous volume (NVVV, a ratio between venous and brain volume) in susceptibility-weighted imaging (SWI) exams in patients with sickle cell disease (SCD).

**Materials and Methods:**

We conducted a retrospective study and qualitatively reviewed for hypointense arterial vessel contamination in SWI exams from 21 children with SCD. We developed a postprocessing framework using magnetic resonance angiography in combination with SWI to provide a more accurate quantification of NVVV. NVVV was calculated before and after removing arterial vessel contributions to determine the error from hypointense arterial vessels in quantifying NVVV.

**Results:**

Hypointense arterial vessel contamination was observed in 86% SWI exams and was successfully corrected by the proposed method. The contributions of hypointense arterial vessels in the original SWI were significant and accounted for approximately 33% of the NVVV [uncorrected NVVV = 0.012 ± 0.005 versus corrected NVVV = 0.008 ± 0.003 (mean ± SD), *P* < 0.01].

**Conclusion:**

Hypointense arterial vessel contamination occurred in the majority of SWI exams and led to a sizeable overestimation of the visible venous volume. A prospective longitudinal study is needed to evaluate if quantitation of NVVV was improved and to assess the role of NVVV as a biomarker of SCD severity or stroke risk.

## 1. Introduction

Stroke is a devastating complication in children with sickle cell disease (SCD) [1]. Patients with SCD routinely undergo magnetic resonance angiography (MRA) for assessment of the integrity of the arterial component of the cerebrovascular system [2]. An intracranial arterial stenosis places patients with SCD at an elevated risk of developing a silent cerebral infarct or an overt stroke [3]. In addition to the arterial system, the venous vasculature can also be visualized and investigated for abnormalities by susceptibility-weighted imaging (SWI) [4]. SWI is a three-dimensional, velocity-compensated, gradient echo (3D-GRE) technique that is sensitive to paramagnetic substances such as deoxyhemoglobin, blood products, and iron. The conspicuity of venous vessels in SWI results from enhancement of the magnetic susceptibility differences between deoxyhemoglobin in venous vessels and that in the adjacent oxygenated tissue [4]. In addition, SWI can detect pathophysiologic changes associated with occluding thrombus and hypoperfused regions of the at-risk tissue, which can be clinically useful in the diagnosis and management of stroke [5–8]. Therefore, accurate quantification of venous conspicuity, which reflects the changing venous flow physiology, is essential to establish a reliable biomarker for the management of stroke risk.

A recent evaluation of SWI found lower venous conspicuity in children with SCD than in healthy controls [9]. The sickle cell study also found that in some patients, irregular or tortuous arteries appeared hypointense on SWI [9]. Reduced SWI venous contrast has been reported in high-flow conditions during anesthesia and carbogen challenges [10, 11]. The hypointense arterial signal emerged from the original magnitude images, which suggests poor performance of the linear flow compensation. Nonlinear or higher-order flow terms could arise from hyperemia, which is characteristic of SCD, and/or from the rapidly changing path of the blood through the tortuous arterial vessels [12, 13]. Importantly, however, the mixed vasculature will result in the overestimation of venous vessel volume if not properly accounted for.

In this study, we propose a postprocessing framework to remove the contamination of hypointense arterial vessels in SWI exams. Removal of the arterial vessels should increase SWI accuracy and interpretation and improve quantification of venous conspicuity. The accurate quantification of venous conspicuity is essential to investigate the potential use of SWI as a biomarker of disease severity or the risk of stroke in patients with SCD.

## 2. Material and Methods

### 2.1. Patients and Study Design

We conducted a retrospective review of diagnostic SWI scans from 21 patients with SCD, all with hemoglobin SS (12 female, mean age 12.9 ± 3.7 years; 9 male, mean age 12.3 ± 3.9 years). Our institutional review board (IRB) approved the study. Twelve exams were performed on 1.5T scanners (Siemens Avanto and Symphony) and 9 exams on a 3T scanner (Siemens Trio). Data were pooled from 1.5T and 3T since a previous study found no field strength difference in NVVV for sickle cell patients [14]. The SWI acquisition was a 3D T2^∗^-weighted gradient-echo sequence with the following parameters (3T/1.5T): TE = 25/40 ms, TR = 56/60 ms, FA = 20°, slice thickness = 2.0 mm, matrix size = 384 × 257 × 72 mm^3^, FOV = 210 × 210 × 144 mm^3^, and an integrated parallel acquisition technique (IPAT) acceleration factor of 2. A sliding minimum intensity projection (mIP, 16 mm thick) was calculated from the SWI. The exam also included a 3D time-of-flight (TOF) MRA optimized for high flow with the following parameters: TE = 4.25 ms, TR = 40 ms, FA = 25°, slice thickness = 0.80 mm, matrix size = 512 × 512 × 82 mm^3^, FOV = 210 × 210 × 66 mm^3^, and IPAT = 2.

### 2.2. Qualitative Analysis

A neuroradiologist with 16 years of experience qualitatively assessed the frequency and degree of hypointense arterial contributions above the M1 segments of the middle cerebral artery (MCA) on the mIP SWI. Examinations were assigned a qualitative score from 0 to III to reflect the degree of arterial vessel contribution: 0, no hypointense arterial vessel contribution within the Sylvian fissure; I, few arterial vessels in the anterior Sylvian fissure; II, arterial vessels extending through to the posterior temporal lobe; and III, multiple tertiary arterial branches in the Sylvian fissure.

### 2.3. Quantitative Analysis

The first step in quantifying the apparent venous contrast in the mIP SWI was brain extraction by using the Brain Extraction Tool (http://www.fmrib.ox.ac.uk/fsl) [15]. From the mIP SWI brains, hypointense tubular structures were segmented by using a 2D Frangi vesselness filter [16] in MATLAB (MathWorks, Natick, MA) with filter parameters of *β* = 0.5 and *c* = 20. This method of vessel segmentation has been previously reported [16–19]. The normalized visible venous volume (NVVV) was calculated by dividing the volume of venous vessels with probability > 60% above the M1 segment of the MCA by the total intracranial volume above the M1 segment of the MCA [9]. NVVV is a dimensionless ratio and is hereafter referred to as uncorrected NVVV (uNVVV) because of the inclusion of any hypointense venous or arterial vessels.

**Figure 1:**
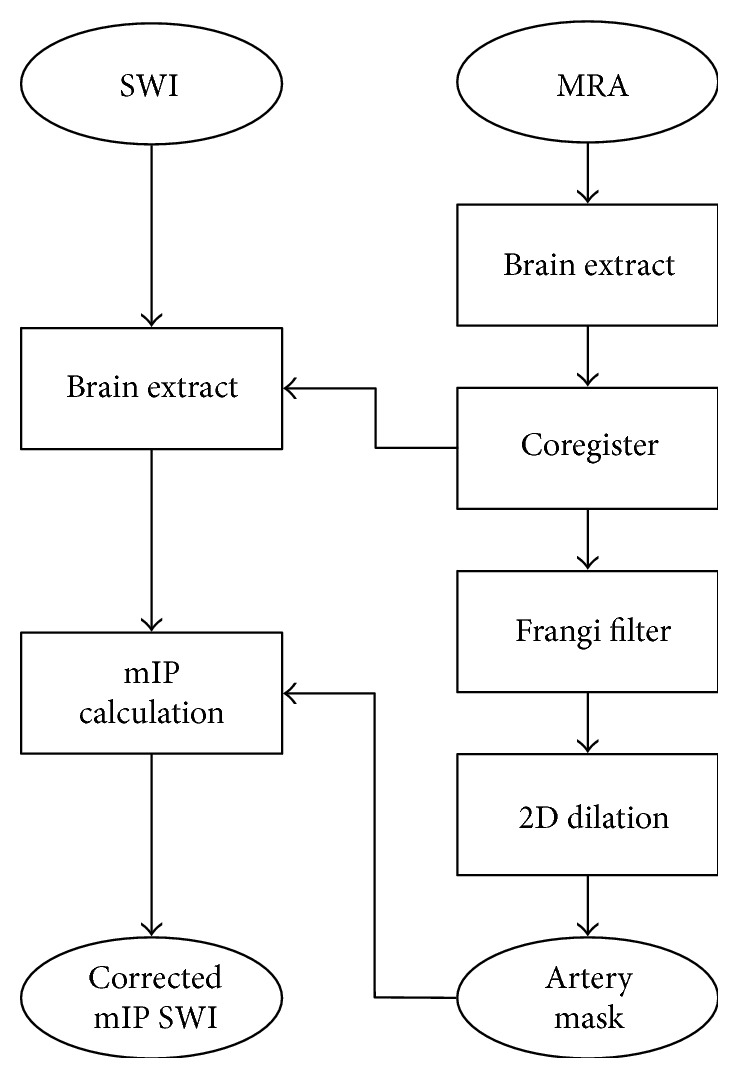
Diagram of the post processing workflow to remove hypointense arterial vessels from SWI images for improved quantification of venous conspicuity. SWI: susceptibility-weighted imaging; mIP: minimum intensity projection; MRA: magnetic resonance angiography.

Figure 1() outlines the method of removing arterial vessel contributions from SWI. A realigned MRA is required for identifying the spatial location of arterial vessels in SWI images, which was done by using normalized mutual information using SPM8 (http://www.fil.ion.ucl.ac.uk/spm). The quality of the coregistration was visually inspected. The same 2D Frangi filter (*β* = 0.5 and *c* = 20) was then used to segment the hyperintense arterial vessels from the realigned MRA. Note that the Frangi vesselness filter can be used to segment hypointense or hyperintense tubular structures according to the sign of the second eigenvalue (hypointense = positive; hyperintense = negative). An arterial mask was created by selecting voxels with a probability of >75% and was dilated by 2 voxels (0.82 mm) in-plane. The optimal probability threshold was empirically determined at the beginning of the study. 75% was the threshold which visually best represented arterial vessels and did not include background voxels from the underlying MRA images. The mask dilatation was applied to minimize any vessel misalignment between the MRA and SWI. The dilatation size was also determined in preliminary studies: 2 voxels provided an optimum to account for partial volume effects and slight misregistration while ensuring that not too much vein volume was excluded. A new corrected 16 mm sliding mIP SWI was calculated after excluding any signal identified as an arterial vessel from the arterial mask. If the projection volume contained the arterial mask only, which is possible when an artery is oriented parallel to the projection volume, the result was chosen to reflect a hyperintense signal intensity. The method describing the calculation of the uNVVV was applied to segment the remaining hypointense structures from the mIP SWI and calculate a corrected NVVV (cNVVV). Repeated-measures ANOVA was applied to test for differences between uNVVV and cNVVV with *P* < 0.05 considered significant.

## 3. Results

The qualitative review revealed hypointense arterial signals in the mIP SWI in 18 of 21 (86%) exams, which had scores of I (*n* = 7), II (*n* = 9), and III (*n* = 2). The subsequent radiological review confirmed both that hypointense signals in the mIP SWI suspected to be arteries agreed well with hyperintense arterial signal from the realigned maximum intensity projection (MIP) TOF images and the grading scores. However, not all arteries in the MIP TOF appeared hypointense on the mIP SWI, only large vessels with anticipated high, nonlinear flow components that could not be compensated by the flow compensation gradients of the SWI sequence. The most prominent pattern of arterial vessel contamination was observed in the anterior Sylvian fissure extending through to the posterior temporal lobe.

**Figure 2:**
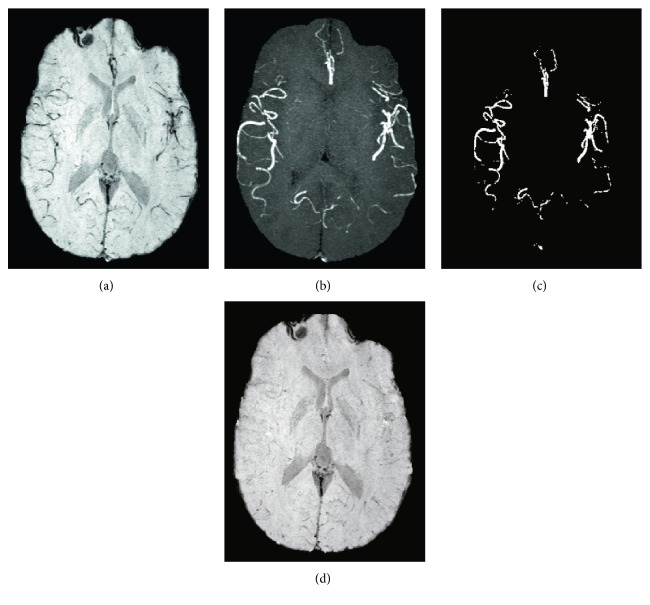
Representative mIP SWI (a) from a patient with SCD showing several hypointense vessels that could be interpreted as veins (uNVVV = 0.014). The corresponding MIP TOF (b) of the mIP indicates that many of the hypointense vessels on the mIP SWI correlate with the hyperintense arterial signal from the TOF. A MIP of the corresponding artery mask was created by the 2D Frangi filter (c). The corrected mIP SWI (d) shows the effectiveness of arterial signal removal (cNVVV = 0.007).

Coregistration was successful in all cases as verified by visual assessment. The framework for removing hypointense arterial vessel contamination in SWI requires accurate arterial vessel segmentation of the TOF MRA.  Figure 2 depicts a representative example that shows all images acquired and created to generate a corrected mIP SWI. A MIP of the corresponding artery mask created by the 2D Frangi filter, which identifies and segments hyperintense vessels in the TOF, is shown in Figure 2(c). A comparison between the original mIP SWI (Figure 2(a)), MIP TOF MRA (Figure 2(b)), and corrected mIP SWI (Figure 2(d)) showed that the method was effective in removing hypointense arterial vessel contamination in SWI. Qualitatively, the corrected mIP SWI showed very little venous vessel conspicuity indicating that most of the initial hypointense vessel-like structures originated from arteries in patients with SCD.

**Figure 3:**
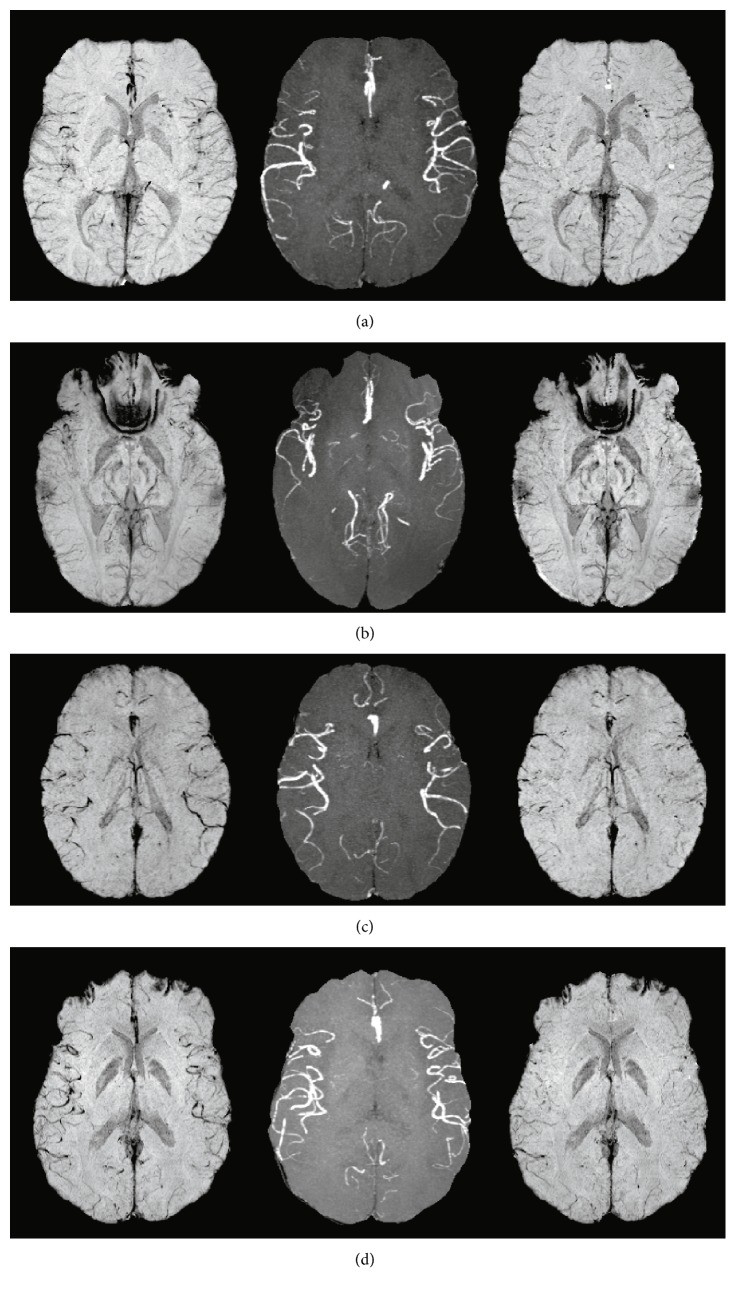
Representative original mIP SWI (left column), MIP TOF (middle column), and corrected mIP SWI (right column) images from the qualitative review showing the degree of arterial vessel contamination (a–d) represent scans from patients with SCD with increasing qualitative scores (scores 0 to III, resp.) of hypointense arterial vessel contamination found in the original mIP SWI. The image in (d) demonstrates that the amount of arterial vessel contamination can be quite large. Note the change in the number of hypointense vessels between the corrected and original mIP SWI.

Figure 3 shows representative patient examples for each degree of arterial vessel contamination from the qualitative review (scores 0–III). The removal of arterial hypointensities from the original mIP SWI was accomplished for all degrees of contamination, demonstrating an effective approach independent of the score.

**Figure 4:**
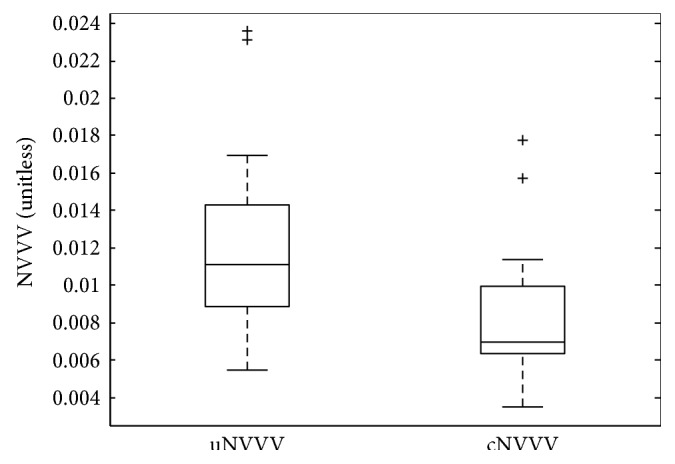
Box plot of the normalized venous vessel volume (NVVV) in 21 patients with SCD before [uncorrected NVVV (uNVVV) = 0.012 ± 0.005 (mean ± SD)] and after arterial signal correction [corrected NVVV (cNVVV) = 0.008 ± 0.003 (mean ± SD)].

The quantitative analysis of NVVV revealed that contributions of hypointense arterial vessels that were confirmed by the radiologist's review in the original mIP SWIs were significant in this cohort of patients with SCD and accounted for approximately 33% of the NVVV [uNVVV = 0.012 ± 0.005 versus cNVVV = 0.008 ± 0.003 (mean ± SD), *P* < 0.01] (Figure 4).

**Figure 5:**
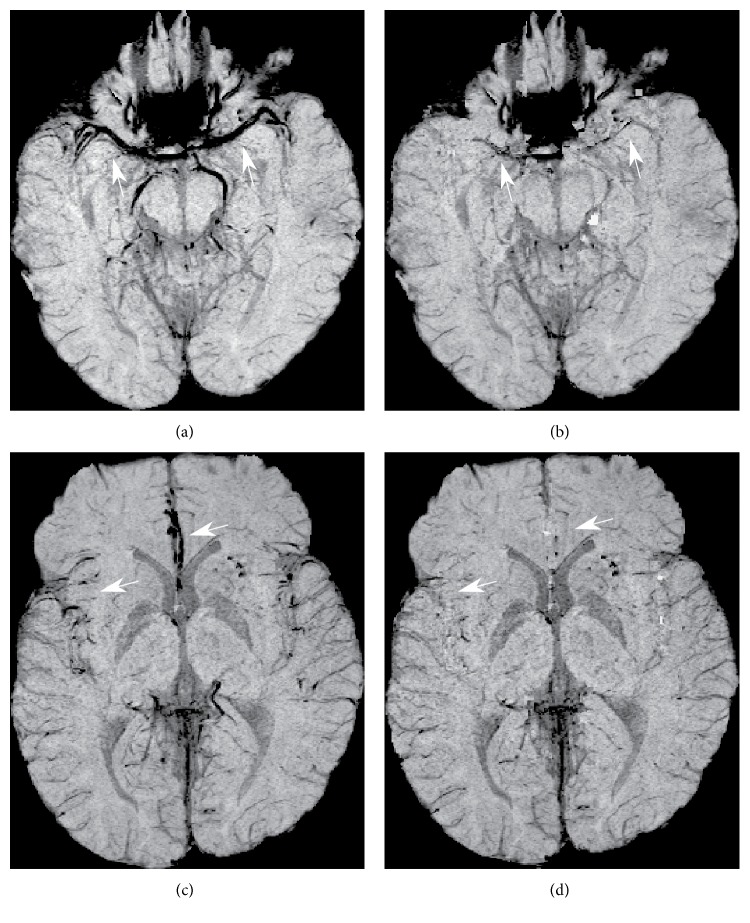
Comparison of the original mIP SWI (a) with the corrected mIP SWI (b) illustrates incomplete removal of the hypointense M1 segment of the MCA as indicated by the arrows. However, in the more superior portion of the brain, hypointense arterial vessels found in the Sylvian fissure and ACA (c) were removed in the corrected mIP SWI (d) as illustrated by the arrows.

The performance of the proposed framework for removing hypointense arterial vessel contamination was qualitatively reviewed again by the same radiologist to confirm suppression of hypointense arterial signals in the mIP SWI. The review showed that hypointense arterial vessels were removed in 20 of 21 mIP SWI exams. Figures 5(a) and 5(b) illustrate the one exceptional exam with incomplete removal of the M1 segment of the MCA. The realignment performance of the MRA with the SWI was sufficient to remove hypointense arterial vessels in more superior portions of the brain as found in the Sylvian fissure and the ACA in the corrected mIP SWI (Figures 5(c) and 5(d)).

## 4. Discussion

In this study, we demonstrate a simple yet effective framework to remove hypointense arterial vessels from SWI exams by using MRA images for quantification of venous conspicuity. Our method measured significant volume contributions from hypointense arterial vessels in the NVVV of patients with SCD; without artery correction, the simple interpretation of any hypointense tubular structures as venous vessels in SWI exams could lead to misclassification of the venous vasculature and overestimation of the visible venous volume.

Semiquantitative analysis of SWI is documented as being valuable for pediatric stroke risk assessment, and SWI hypointense venous signal was correlated with stroke progression [20]. In a cohort of patients with SCD, it was observed that the disease can affect SWI contrast by producing globally diminished venous conspicuity and attempts to quantify this observation via NVVV analysis were undertaken [9]. While previously no correlation of hemoglobin and other laboratory variables with NVVV was demonstrated, the explanation is likely complex and possibly related in part to decreased concentration of deoxyhemoglobin and elevated cerebral blood flow, although this would need to be tested in a prospective study. The isointense venous presentation seen in our cohort resembles the contrast seen in high-blood-flow conditions induced through carbogen challenges and sedation [10, 11]. The investigation of possible physiologic mechanisms underlying decreased venous conspicuity in order to establish its association with the risk of stroke in patients with SCD requires the quantification of venous contrast as accurately as possible. The presented method is a step forward towards meeting this goal by eliminating potential errors in the NVVV quantification introduced by misclassification of arteries as veins. However, as no ground truth for *in vivo* NVVV values exists, we cannot perform accuracy tests and hence we can only claim based on the radiologist's review and logical deduction that the NVVV quantification process was indeed improved.

In our study, we observed that 86% of SCD SWI exams contained a mixture of arterial and venous vasculature. Hypointense arterial vessels were observed not only in brain regions ranging from the anterior Sylvian fissure and extending to the posterior temporal lobe but also in portions of the anterior and posterior cerebral arteries. The arterial vessels appeared tortuous, which is characteristic of cerebral arteries in patients with SCD [13, 21]. The changing flow dynamics of blood through the tortuous arteries could contribute to nonlinear or higher-order flow terms such as acceleration, which result in signal dephasing and a hypointense arterial signal. An advanced SWI sequence that incorporates both constant and accelerated flow compensation could potentially also reduce arterial vessel contamination [22]. The retrospective nature of our study did not allow testing of a modified SWI sequence in patients with SCD. However, our method attempts to correct the errors in NVVV quantification based on imperfect SWI acquisition. The quantification of venous contrast before and after arterial vessel removal by our method suggested that approximately one-third of the apparent venous volume by SWI was arterial. This finding indicates that appropriate methods must be implemented during image acquisition or postprocessing to eliminate the contribution of hypointense arterial vessels in order to calculate the NVVV.

To separate hypointense venous and arterial vessels in the SWI, our method used TOF MRA to identify the anatomic locations of arterial vessels. TOF MRA is a routine and robust imaging method to study arterial vasculature. Because MRA is usually a part of the routine clinical MR exam of patients with SCD to screen for stenosis and help assess the risk of stroke, our method will not require any additional exam time in most cases.

Our framework of removing hypointense arterial vessels used the same segmentation method for both MRA and SWI. An important consideration in applying the arterial mask is that it will not affect the visualization of venous vessels in the mIP SWI, which would result in an underestimation of the visible venous volume. Arterial vessel masking was performed in the original SWI space before the mIP calculation to avoid the masking of any venous vessels in the larger projection volume (16 mm). Applying an arterial mask directly to the mIP SWI will lead to underestimation of the NVVV, because it would not account for the venous vessels masked by the arteries in the projection. Further, it has to be noted that dilatation could also potentially lead to an underestimation of the NVVV: a dilatation factor which is chosen too large may disguise veins that are running in parallel and proximity to the dilated arteries. With a carefully chosen dilatation factor, however, this error can be minimized. On the other hand, without dilatation, residual arterial signal remains most likely due to partial volume effects and minor misregistration, so a balance needs to be struck.

Our proposed method of arterial vessel removal has some limitations. The success of the method primarily depends on the quality and coverage of the MRA. Arterial vessel enhancement in MRA is important and is limited to flow-related enhancement. The MRA needs be optimized for high flow in SCD patients. Because MRA coverage should include all the areas of NVVV quantification, it may be necessary to increase the coverage or reposition of the MRA to include more superior portions of the brain. Incomplete overlap of the MRA with areas of NVVV quantification could result in arterial vessels not included in the mask, which could lead to an overestimation of NVVV. Furthermore, motion artifacts in the MRA and SWI will limit the effectiveness of the Frangi filter to segment vessels by reducing the vessel probability below the cutoff threshold. For MRA, motion will result in an incomplete arterial mask and overestimation of NVVV. For SWI, incomplete vessel segmentation will result in an underestimation of NVVV. Another limitation is the small sample size of this study that did not allow for advanced statistical analyses such as gender and field strength differences in NVVV for SCD patients. This should be addressed in future prospective SCD imaging studies.

## 5. Conclusion

We found that a large percentage of SCD SWI exams had hypointense arterial vessel contamination. Our proposed postprocessing method identified and removed arterial vessel contamination in SWI exams, thereby eliminating one source of error in the estimation of venous vessel conspicuity in patients with SCD. This method of quantifying venous conspicuity can guide future prospective studies on the complex mechanisms of SCD and help gain further insight into cerebral hemodynamics in oxygenation and the use of venous conspicuity as a potential biomarker of SCD severity or the risk of stroke.

## References

[1] Ohene-Frempong K., Weiner S. J., Sleeper L. A. (1998). Cerebrovascular accidents in sickle cell disease: rates and risk factors. *Blood*.

[2] Debaun M. R., Derdeyn C. P., McKinstry R. C. (2006). Etiology of strokes in children with sickle cell anemia. *Mental Retardation and Developmental Disabilities Research Reviews*.

[3] Arkuszewski M., Krejza J., Chen R. (2014). Sickle cell anemia: intracranial stenosis and silent cerebral infarcts in children with low risk of stroke. *Advances in Medical Sciences*.

[4] Reichenbach J. R., Haacke E. M. (2001). High-resolution BOLD venographic imaging: a window into brain function. *NMR in Biomedicine*.

[5] Verma R. K., Hsieh K., Gratz P. P. (2014). Leptomeningeal collateralization in acute ischemic stroke: impact on prominent cortical veins in susceptibility-weighted imaging. *European Journal of Radiology*.

[6] Santhosh K., Kesavadas C., Thomas B., Gupta A. K., Thamburaj K., Kapilamoorthy T. R. (2009). Susceptibility weighted imaging: a new tool in magnetic resonance imaging of stroke. *Clinical Radiology*.

[7] Hermier M., Nighoghossian N. (2004). Contribution of susceptibility-weighted imaging to acute stroke assessment. *Stroke*.

[8] Greer D. M., Koroshetz W. J., Cullen S., Gonzalez R. G., Lev M. H. (2004). Magnetic resonance imaging improves detection of intracerebral hemorrhage over computed tomography after intra-arterial thrombolysis. *Stroke*.

[9] Winchell A. M., Taylor B. A., Song R. (2013). Evaluation of SWI in children with sickle cell disease. *AJNR - American Journal of Neuroradiology*.

[10] Sedlacik J., Kutschbach C., Rauscher A., Deistung A., Reichenbach J. R. (2008). Investigation of the influence of carbon dioxide concentrations on cerebral physiology by susceptibility-weighted magnetic resonance imaging (SWI). *NeuroImage*.

[11] Sedlacik J., Lobel U., Kocak M. (2010). Attenuation of cerebral venous contrast in susceptibility-weighted imaging of spontaneously breathing pediatric patients sedated with propofol. *AJNR - American Journal of Neuroradiology*.

[12] Ausavarungnirun P., Sabio H., Kim J., Tegeler C. H. (2006). Dynamic vascular analysis shows a hyperemic flow pattern in sickle cell disease. *Journal of Neuroimaging*.

[13] Steen R. G., Reddick W. E., Glass J. O., Wang W. C. (1998). Evidence of cranial artery ectasia in sickle cell disease patients with ectasia of the basilar artery. *Journal of Stroke and Cerebrovascular Diseases*.

[14] Winchell A. M., Taylor B. A., Song R. (2014). Evaluation of SWI in children with sickle cell disease. *AJNR - American Journal of Neuroradiology*.

[15] Smith S. M. (2002). Fast robust automated brain extraction. *Human Brain Mapping*.

[16] Frangi A., Niessen W., Vincken K., Viergever M., Wells W., Colchester A., Delp S. (1998). Multiscale vessel enhancement filtering. *Medical Image Computing and Computer-Assisted Interventation — MICCAI’98*.

[17] Sato Y., Nakajima S., Shiraga N. (1998). Three-dimensional multi-scale line filter for segmentation and visualization of curvilinear structures in medical images. *Medical Image Analysis*.

[18] Koopmans P. J., Manniesing R., Niessen W. J., Viergever M. A., Barth M. (2008). MR venography of the human brain using susceptibility weighted imaging at very high field strength. *Magma*.

[19] Zivadinov R., Poloni G. U., Marr K. (2011). Decreased brain venous vasculature visibility on susceptibility-weighted imaging venography in patients with multiple sclerosis is related to chronic cerebrospinal venous insufficiency. *BMC Neurology*.

[20] Polan R. M., Poretti A., Huisman T. A., Bosemani T. (2015). Susceptibility-weighted imaging in pediatric arterial ischemic stroke: a valuable alternative for the noninvasive evaluation of altered cerebral hemodynamics. *AJNR - American Journal of Neuroradiology*.

[21] Steen R. G., Langston J. W., Ogg R. J., Manci E., Mulhern R. K., Wang W. (1998). Ectasia of the basilar artery in children with sickle cell disease: relationship to hematocrit and psychometric measures. *Journal of Stroke and Cerebrovascular Diseases*.

[22] Pipe J. G., Chenevert T. L. (1991). A progressive gradient moment nulling design technique. *Magnetic Resonance in Medicine*.

